# An Overview of the Diagnostic and Treatment Strategies for Aortic Disease

**DOI:** 10.31083/RCM47202

**Published:** 2026-04-20

**Authors:** Jianwen Xu, Fujian Wang, Kai Wang, Shu Zhang

**Affiliations:** ^1^Department of Emergency Medicine, West China Hospital, Sichuan University, 610041 Chengdu, Sichuan, China

**Keywords:** aorta, aortic disease, acute aortic syndrome, aneurysms, thoracic aortic aneurysms

## Abstract

The aorta, a vital conduit that transports oxygenated blood from the heart to the systemic circulation, is characterized by intricate architecture and heterogeneous embryological origins. Recently, the aorta has been conceptualized as a functionally integrated “aortic organ”, providing a comprehensive framework that supports the systematic evaluation, management, and long-term surveillance of aortic pathologies. Aortic disease primarily encompasses acute aortic syndromes and chronic aneurysmal disorders, which are often characterized by asymptomatic onset and rapid clinical progression, posing a significant risk of mortality in the absence of prompt diagnosis and intervention. This review provides a systematic overview of the classification, epidemiological features, diagnostic approaches, and therapeutic advances in aortic diseases. Moreover, this review outlines current indications, technical considerations, and clinical outcomes associated with various treatment strategies. Finally, this review identifies key directions for future research, including standardizing diagnostic classifications, refining risk-stratification models, and advancing comprehensive endovascular therapies, with the ultimate goal of enhancing lifelong patient management and improving clinical outcomes.

## 1. Introduction

**Fig. 1.**
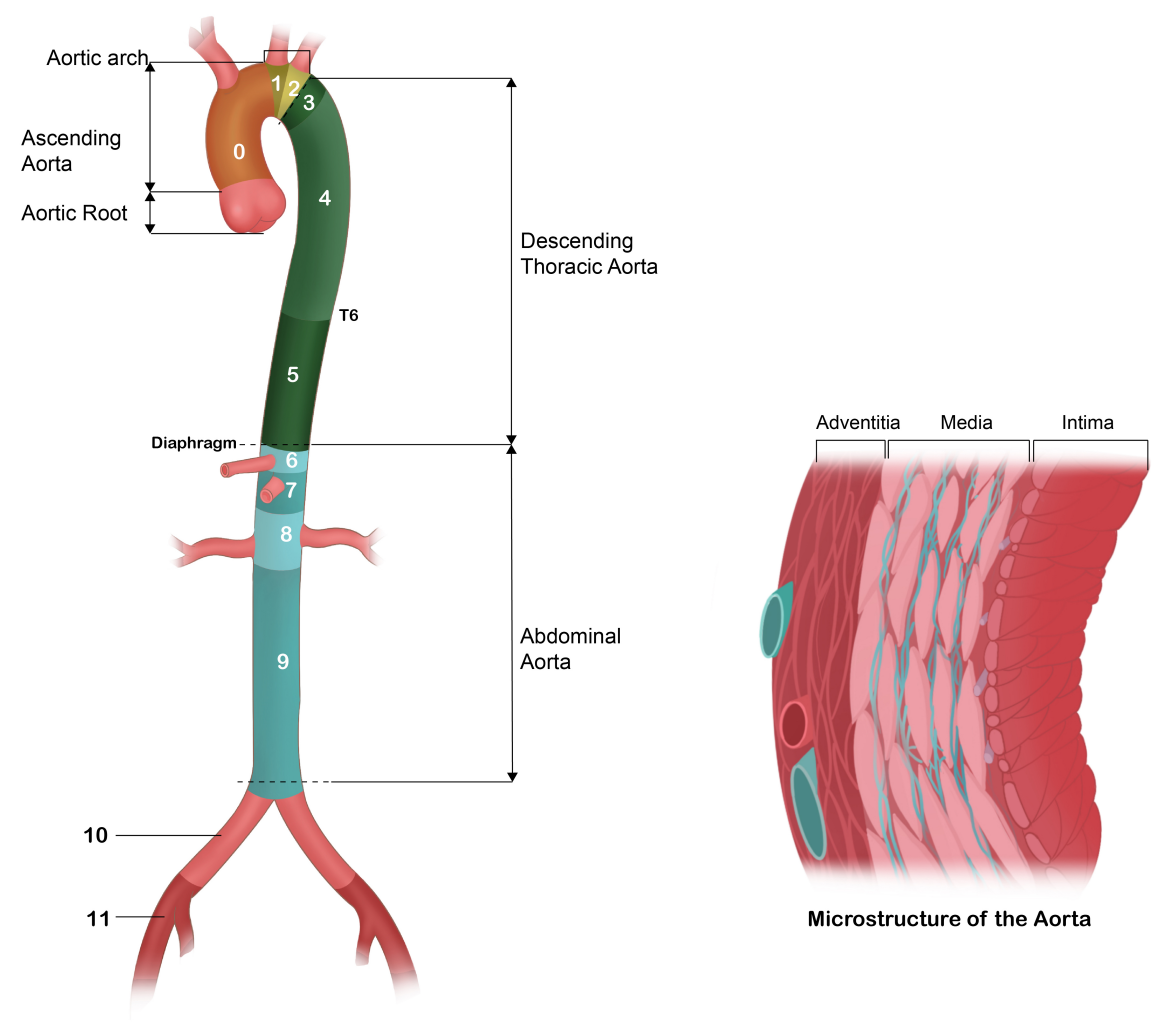
**Aortic segments and Ishimaru zones**.

The aorta serves as the primary conduit for delivering oxygenated blood from the heart to peripheral organs. Due to its functional integration, it has been increasingly recognized that the aorta should be conceptualized, evaluated, and managed as a unified organ system. Accordingly, its diagnosis, treatment, and long-term surveillance must be guided by this integrative perspective [1]. The aorta also demonstrates heterogeneous developmental origins: the aortic root arises from heart field cells, the ascending aorta and proximal aortic arch originate from neural crest cells, whereas more distal segments are derived from mesodermal precursors [2]. Anatomically, the aorta is divided into five major segments (Fig. 1). The Ishimaru classification further refines this segmentation by dividing the aorta into 12 distinct zones (0–11), providing a standardized anatomical framework that is critical for the precise characterization and clinical management of aortic dissections and aneurysms [3].

Aortic diseases (AD) are broadly classified into acute aortic syndromes, chronic aortic aneurysmal diseases, and several rare entities. These conditions involve structural or functional impairment of the aortic wall, compromising its ability to withstand hemodynamic forces. This vulnerability predisposes to serious complications, including dilation (aneurysm), intimal tearing (dissection), rupture, or stenosis. Without prompt intervention, acute aortic dissection is associated with a mortality rate that increases by 1–2% per hour during the initial 24–48 hours, culminating in a fatality rate of up to 75% within two weeks of the onset of symptoms [4].

Epidemiological investigations into AD face considerable challenges. First, the frequently asymptomatic preclinical phase and sudden clinical presentation mean that most available data are drawn from symptomatic cohorts, potentially underrepresenting individuals with silent disease. Second, the increased early mortality leads to underdiagnosis in fatal cases, likely resulting in significant underestimation of the true incidence and prevalence. Third, inconsistencies in diagnostic criteria, screening practices, and a lack of consensus on pathological and morphological definitions introduce variability across studies, undermining comparability and contributing to conflicting conclusions. Table 1 (Ref. [4, 5, 6, 7, 8, 9, 10, 11, 12, 13, 14, 15, 16] summarizes key findings from recent epidemiological studies on the incidence and prevalence of AD. The significant geographical variation in the reported incidence of aortic diseases (e.g., 17.6 per 100,000 person-years in Japan vs. 3.47 in Australia) likely stems from multifactorial causes. First, population demographics play a crucial role; countries with rapidly aging populations, such as Japan, naturally exhibit higher rates of aortic pathology. Second, genetic predispositions and lifestyle factors, particularly salt intake and the prevalence of hypertension, vary markedly across regions. Finally, methodological differences significantly influence these rates. For instance, the notably higher incidence reported in Japanese studies stems largely from the inclusion of cases diagnosed at autopsy, which allows for the capture of pre-hospital mortalities (sudden deaths) that are often excluded in strictly clinical registries from other regions.

**Table 1.** **Research on the incidence and prevalence of aortic diseases**.

Period	Country	Disease type	Incidence	Remark
20 y follow	Sweden	Aortic dissections	15 per 100,000 p-y	95% CI (confidence interval) 11.7 to 18.9
		Thoracic aortic aneurysms	9.0 per 100,000 p-y	95% CI 6.8 to 12.6
		Abdominal aortic aneurysms	27 per 100,000 p-y	95% CI 22.5 to 32.1 [5]
Not specified	Iran	Thoracic aorta aneurysm	1.2%	Prevalence rate [6]
2000–2008	Italy	Aortic dissections	4.7 per 100,000 p-y	Davide Pacini *et al*. [7]
2002–2012	UK	Acute aortic dissection	6 per 100,000 p-y	[8]
2002–2016	Sweden	Acute aortic dissection	7.2 per 100,000 p-y	[9]
2005–2012	China	Acute aortic dissection	5.6 per 100,000 p-y	[10]
2006–2016	Korea	Aortic dissection	3.76 per 100,000 p-y	[11]
2017–2018	Australia	Aortic dissection	3.47 per 100,000 p-y	[12]
2016–2018	Japan	Aortic dissection	17.6 per 100,000 p-y	Including the autopsy results [13]
1996–2016	Denmark	Aortic dissection	4.2 per 100,000 p-y	[14]
1995–2015	America	Aortic dissection	4.4 per 100,000 p-y	[4]
		Penetrating aortic ulcer	2.1 per 100,000 p-y	
		Intramural hematoma	1.2 per 100,000 p-y	
2006–2014	Sweden	Abdominal aortic aneurysm	1.5%	Prevalence rate
				For 65-year-old men [15]
1990–2015	UK	Abdominal aortic aneurysm	dropped from 5% to 1.3%	Prevalence rate
				For 65-year-old men [16]

This review offers a comprehensive overview of AD, with a primary focus on aortic aneurysms and acute aortic syndromes. We outline current diagnostic strategies and advanced imaging modalities, summarize established and emerging treatment approaches, discuss surveillance protocols, and highlight directions for future research, which are essential for advancing the understanding and management of aortic pathologies.

## 2. Classification of Aortic Diseases

### 2.1 Acute Aortic Syndrome (AAS)

**Fig. 2.**
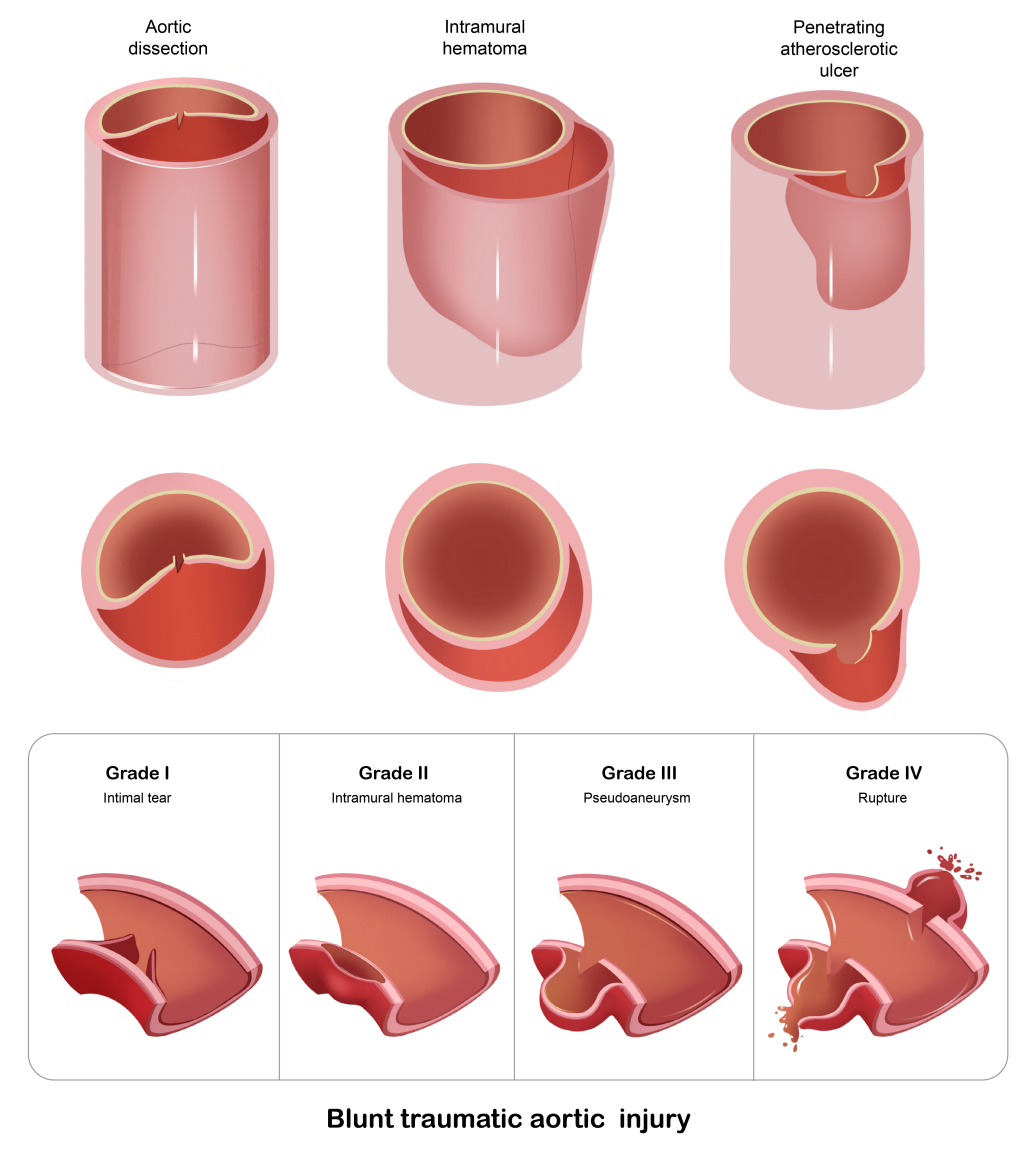
**The types of acute aortic syndrome and the classification of blunt traumatic aortic injury**.

AAS encompasses a spectrum of life-threatening conditions arising from pathological alterations in the aortic wall [17]. This clinical entity includes aortic dissection, intramural hematoma, penetrating atherosclerotic ulcer, and traumatic aortic injury, all of which pose a significant risk for aortic rupture (Fig. 2).

#### 2.1.1 Aortic Dissection

**Fig. 3.**
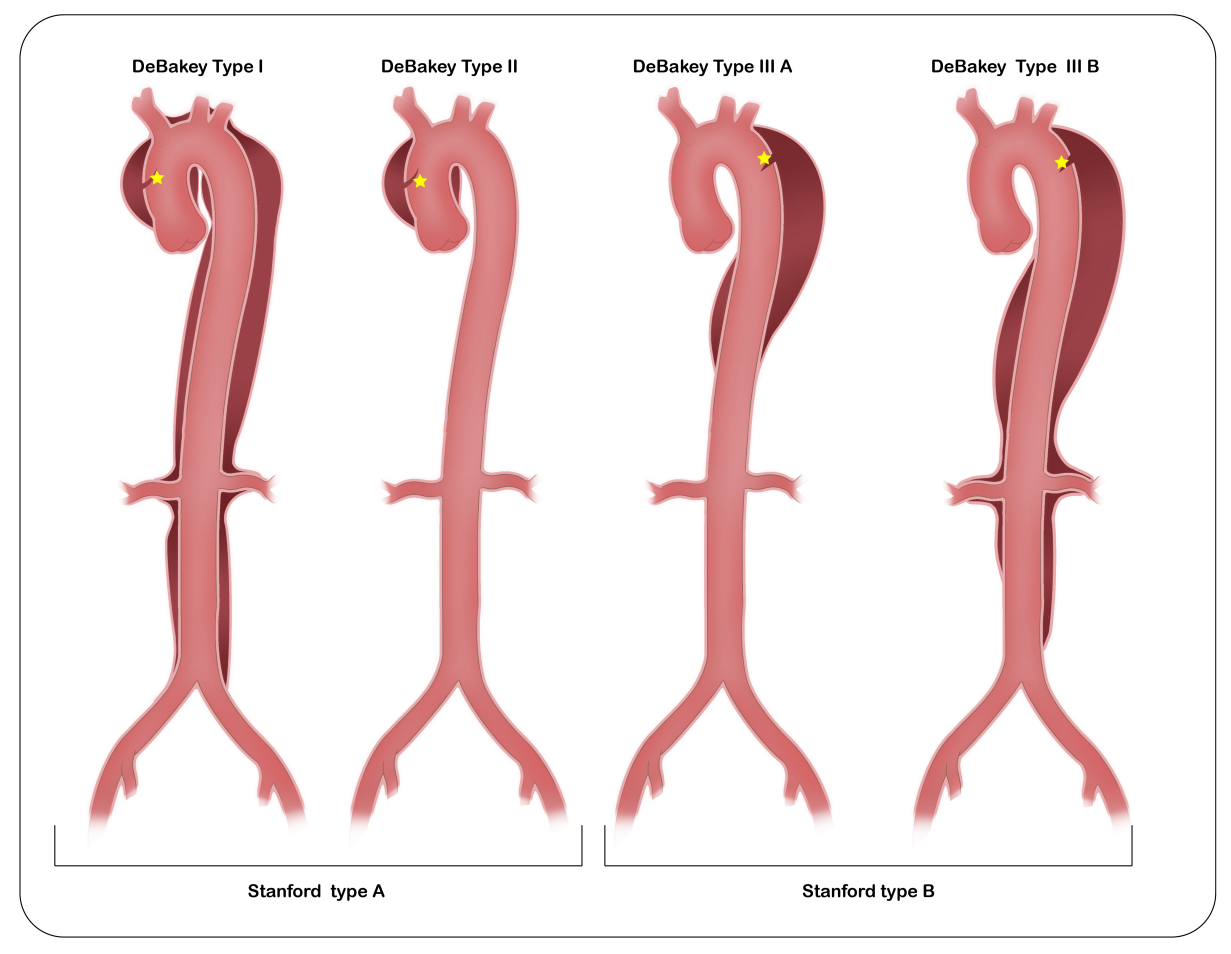
**Stanford and DeBakey classification of acute aortic dissection**.

Aortic dissection represents the most prevalent form of AAS. The aortic wall is composed of three distinct layers: the intima, media, and adventitia. Aortic dissection is an acute pathological process initiated by a tear in the intimal layer, leading to separation between the intima and media. This results in the formation of a secondary channel—the false lumen—adjacent to the native vessel lumen, referred to as the true lumen. According to the 2020 consensus report by the Society for Vascular Surgery (SVS) and the Society of Thoracic Surgeons (STS), aortic dissections should be categorized into four temporal phases: hyperacute (<24 hours), acute (1–14 days), subacute (15–90 days), and chronic (>90 days). This classification aims to enhance prognostic accuracy and inform clinical decision-making regarding the timing and type of potential interventions [18]. Two widely adopted anatomical classification systems are the DeBakey and Stanford classifications (Fig. 3). Regardless of the site of the primary intimal tear, involvement of the ascending aorta defines a Stanford Type A dissection (corresponding to DeBakey Types I and II), whereas dissections confined to the descending aorta are classified as Stanford Type B (DeBakey Types IIIa or IIIb) [19]. In 2019, a joint expert consensus statement from the European Association for Cardio-Thoracic Surgery (EACTS) and the European Society for Vascular Surgery (ESVS) introduced an additional category—“non-A-non-B dissection”—to describe cases involving the aortic arch without extension into the ascending aorta. Subsequently, in 2020, the SVS and STS proposed a refined classification system that provides greater anatomical detail by categorizing dissections based on the location of the intimal tear and the proximal and distal extent of the dissection [18]. A European adaptation of the Stanford classification—the Type/Entry location/Malperfusion classification—has recently been introduced [20]. Although no single classification system has yet demonstrated definitive superiority in predicting patient outcomes or guiding therapeutic options, emerging guidelines may facilitate more precise individualized assessment. Future research should focus on standardizing dissection classification to improve clinical consistency and comparability across studies.

#### 2.1.2 Intramural Hematoma (IMH)

IMH is defined by the presence of a localized hemorrhage exceeding 5 mm in thickness within the aortic wall, which may occur in the absence or presence of an identifiable intimal tear [21]. IMH is classified as Type A when the ascending aorta is involved and as Type B when it is limited to the descending aorta. It is commonly regarded as a precursor or atypical variant of a classic aortic dissection and typically warrants similar management strategies, particularly in Type A cases. Diagnosis is primarily obtained through computed tomography angiography (CTA), magnetic resonance imaging (MRI), or echocardiography, with imaging findings characterized by circumferential or crescent-shaped thickening of the aortic wall exceeding 5 mm and the absence of detectable blood flow within the hematoma. The natural course of IMH is heterogeneous. Approximately 10% of cases resolve spontaneously, whereas 16% to 47% may progress to an overt aortic dissection if the intimal layer ruptures and establishes a communication with the true lumen [22].

#### 2.1.3 Penetrating Atherosclerotic Ulcer (PAU)

A penetrating atherosclerotic ulcer is characterized by an ulcerated atherosclerotic plaque that disrupts the internal elastic lamina and extends into the medial layer of the aortic wall. Most PAUs are asymptomatic and are incidentally detected during imaging performed for unrelated indications; they are predominantly located in the descending thoracic aorta [23]. Their exact incidence remains uncertain but is estimated to constitute 2% to 7% of all acute aortic syndromes [24].

#### 2.1.4 Traumatic Aortic Injury

Aortic transection resulting from penetrating trauma is typically associated with active, life-threatening hemorrhage, necessitating immediate surgical intervention in nearly all cases. In contrast, blunt traumatic aortic injury primarily arises from rapid deceleration forces, which induce differential movement between mobile and fixed segments of the aorta. Blunt thoracic aortic injury (BTAI) most frequently occurs at the aortic isthmus, although less common sites have been reported [25]. Clinical presentation varies widely depending on the severity of the injury, ranging from asymptomatic or non-specific chest pain to hemorrhagic shock. BTAI is classified into four grades: Grade I, intimal tear; Grade II, intramural hematoma; Grade III, pseudoaneurysm; and Grade IV, free rupture.

### 2.2 Chronic Aortic Aneurysmal Diseases

#### 2.2.1 Thoracic Aortic Aneurysm (TAA)

The incidence of TAA ranges from 5 to 10 cases per 100,000 person-years [26]. Among all TAAs, aneurysms involving the aortic root, ascending aorta, or both are the most prevalent, accounting for approximately 60% of cases, followed by those affecting the descending thoracic aorta (~30%) and the aortic arch (<10%). Major risk factors for the development of a TAA include hypertension, smoking, hypercholesterolemia, and genetic predisposition.

Aortic Root and Ascending Aorta: The conventional definition of an aneurysm as any dilation exceeding 1.5 times the expected normal diameter is well established for the abdominal and descending thoracic aorta but appears less applicable to the aortic root and ascending segment [25]. Evidence suggests that an ascending aortic diameter greater than 4.0 cm may be considered dilatation, whereas a diameter exceeding 4.5 cm is more consistently associated with aneurysmal pathology [21, 27]. Given individual variability in body size, particularly among taller individuals, aortic dimensions should be indexed to body surface area or height. The aortic size index or the simpler aortic height index are commonly used for normalization, both of which demonstrate superior prognostic value for adverse outcomes compared to the absolute diameter alone [28, 29]. Furthermore, the ratio of aortic cross-sectional area to patient height has emerged as a valid risk stratification tool, with specific thresholds strongly correlated with increased risk of rupture or dissection [30]. These individualized, size-adjusted metrics are essential for advancing precision medicine in the management of aortic disease.

Aortic Arch: The aortic arch extends from the brachiocephalic trunk to the left subclavian artery. Pathologies of the arch frequently arise as chronic sequelae following surgical repair of Type A aortic dissection (TAAD) [1]. Isolated aortic arch aneurysms are uncommon, representing only about 10% of all TAAs [31]. In addition to pain or pressure-like sensations, arch aneurysms may produce symptoms through compression of adjacent mediastinal structures, including dyspnea, cough, or recurrent laryngeal nerve palsy leading to vocal cord paralysis [32].

Descending Aorta: Morphologically, descending TAAs differ markedly from their ascending counterparts. Ascending TAAs are typically smooth-walled, non-calcified, not directly linked to atherosclerosis, and devoid of intraluminal thrombus, whereas descending TAAs often exhibit calcification, luminal irregularity, and thrombus formation—features consistent with atherosclerotic degeneration [33]. These phenotypic differences are partly attributed to distinct embryological origins: the smooth muscle cells of the ascending aorta derive from neural crest cells, while those in the descending aorta originate from mesodermal precursors. This fundamental developmental divergence may underlie the differing pathophysiological mechanisms and clinical behavior observed in these segments [34].

#### 2.2.2 Thoracoabdominal Aortic Aneurysm (TAAA)

A thoracoabdominal aortic aneurysm (TAAA) is defined as a continuous aneurysmal dilation spanning both the thoracic and abdominal aorta, crossing the diaphragm. Importantly, it is not simply the coexistence of a thoracic and abdominal aneurysm, but rather a single, contiguous pathological process that involves the aortic hiatus and frequently encompasses the origins of critical visceral arteries—including the celiac axis, superior mesenteric artery, and renal arteries. Due to its extensive anatomical involvement and proximity to vital vascular branches, TAAA repair poses significantly greater technical challenges and higher perioperative risks compared to isolated aneurysms. The Crawford-Safi classification system categorizes TAAAs based on the longitudinal extent of aortic involvement [35]: Extent I: Extends from the distal to the left subclavian artery to the celiac axis or superior mesenteric artery, above the renal arteries. Extent II: Extends from the distal to the left subclavian artery to the infrarenal aorta, often reaching the aortic bifurcation. Extent III: Originates in the mid-descending thoracic aorta (below the 6th intercostal space) and extends to the infrarenal aorta. Extent IV: Begins at the diaphragmatic hiatus (below the 12th thoracic vertebra) and extends to the infrarenal aorta. Extent V: Located below the 6th intercostal space and extends to just above the renal arteries. This classification not only provides a standardized anatomical description but also serves as a robust predictor of operative morbidity and mortality, guiding treatment planning and patient counseling [36].

#### 2.2.3 Abdominal Aortic Aneurysm (AAA)

Abdominal aortic aneurysm (AAA) is the most prevalent form of aortic disease, defined as a localized dilation where the aortic diameter exceeds 1.5 times the expected normal value or reaches an absolute diameter greater than 3.0 cm [37]. The majority of AAAs are asymptomatic and are often detected incidentally during imaging for unrelated conditions. The incidence is fourfold higher in males than in females [38]. While the risk of rupture increases with aneurysm diameter, females face a fourfold higher risk of rupture at any given size, and they also experience higher surgical mortality rates [39]. AAA pathogenesis arises from a complex interplay of genetic and environmental factors, with the most significant contributors being advanced age, male sex, cigarette smoking, and a positive family history [26, 40].

#### 2.2.4 Heritable Aortic Diseases

Marfan Syndrome: An autosomal dominant connective tissue disorder caused by pathogenic variants in the *FBN1* gene, affecting approximately 1 in 5000 individuals [41]. Patients are predisposed to progressive aneurysmal dilation of the aortic root, placing them at high risk for acute aortic dissection. Involvement of the descending and abdominal aorta is less common [41, 42].

Loeys-Dietz Syndrome (LDS): Characterized by widespread arterial aneurysms and dissections, including the aorta and its major branches, along with arterial tortuosity and skeletal features resembling Marfan syndrome. LDS is distinguished by unique craniofacial and cutaneous manifestations [43]. It results from pathogenic variants in genes involved in transforming growth factor-beta (TGF-β) signaling, collectively termed TGF-β vasculopathies. All known LDS-associated genes confer an elevated risk not only for root and ascending aortic disease, but also for branch vessel aneurysms and intracranial aneurysms [21, 43].

Turner Syndrome: A chromosomal disorder occurring in approximately 1 in 2500 live female births, resulting from complete or partial monosomy of the X chromosome [44]. Cardiovascular abnormalities are present in roughly half of the affected individuals, including bicuspid aortic valve (15–30%), coarctation of the aorta (7–18%), and dilatation of the ascending aorta (33%) [44].

Vascular Ehlers-Danlos Syndrome (vEDS): A rare, severe, and frequently life-threatening condition. The vast majority of cases are caused by mutations in the *COL3A1* gene, which encodes the pro-alpha1 chain of type III collagen—a key structural component of blood vessels and hollow organs [45]. vEDS is recognized as the most aggressive subtype of Ehlers-Danlos syndrome due to its high propensity for spontaneous arterial or visceral rupture. It follows an autosomal dominant inheritance pattern, although approximately 50% of cases arise from de novo mutations [46].

#### 2.2.5 Adult Congenital Heart Disease

Bicuspid Aortic Valve (BAV) Aortopathy: BAV is a common congenital valvular anomaly, affecting approximately 1% of the general population, with a male predominance (male-to-female ratio of ~2–3:1) [47]. It is frequently associated with aortic valve dysfunction, including stenosis and regurgitation. Patients commonly develop dilatation or aneurysms of the aortic root, ascending aorta, or both, with the prevalence increasing with age [48]. Recent international expert consensus has established a standardized nomenclature and classification system for BAV and its associated aortopathy: the valve morphology should be described as “fusion-type”, “two-sinus”, or “partial-fusion”; the aortic phenotype is categorized as “Root” (15–20%, predominant sinus dilation), “Ascending” (70–75%, predominant tubular segment dilation), or “Extended” (5–10%, either root dilation extending into the tubular portion or tubular dilation involving the proximal arch) [49].

#### 2.2.6 Chronic Infrarenal Occlusive Aortoiliac Disease

Chronic, extensive occlusive disease of the infrarenal aorta and iliac arteries—commonly known as the Leriche syndrome—is one of the most severe manifestations of large-vessel atherosclerosis [50]. Clinical presentation typically includes exertional claudication characterized by cramping pain in the hips, thighs, and buttocks, accompanied by diminished or absent femoral pulses. However, a subset of patients remains asymptomatic despite a significant disease burden.

### 2.3 Other Aortic Conditions

This category encompasses less common but clinically significant aortic pathologies, including aortitis, endoleaks, aortic infections, aortic atherosclerosis, aortic coarctation, aberrant subclavian artery, and aortic tumors.

Aortitis: Takayasu arteritis and giant cell arteritis are the leading causes of aortitis, both are immune-mediated vasculopathies that can result in aortic aneurysm formation, dissection, IMH, and PAU [51]. Endoleak: Defined as persistent blood flow within the aneurysm sac after endovascular aneurysm repair (EVAR), demonstrated by contrast material outside the stent graft but contained within the sac, indicating incomplete exclusion of the aneurysm from the circulation.

Infectious Aortitis: Refers to infection of the native aortic wall, typically arising from contiguous spread or septic embolization. Common causative organisms include Staphylococcus aureus, Streptococcus pneumoniae, Escherichia coli, and Salmonella species [52, 53]. Syphilitic aortitis, which manifests 10–25 years after the primary infection, is now rare. Fungal (e.g., Candida, Aspergillus) and tuberculous aortitis occur predominantly in immunocompromised individuals [53].

Aortic Atherosclerosis: A chronic immuno-inflammatory and fibro-proliferative disease affecting the aorta and its major branches. Over time, it may result in extensive plaque burden, leading to complications such as aortic thrombosis, occlusion, or severe calcification known as “porcelain aorta” [54].

Aortic Coarctation: A congenital narrowing of the aorta, most frequently located just distal to the left subclavian artery, often accompanied by post-stenotic aneurysmal dilation. It is associated with serious complications, including aortic dissection, aneurysm formation, and refractory hypertension [55].

Aberrant Subclavian Artery: Typically, an incidental finding, as most individuals are asymptomatic. However, a minority may develop symptoms such as dysphagia lusoria or dyspnea due to compression from a retro-esophageal course [56]. Dilation at the origin of the aberrant vessel forms a Kommerell diverticulum, which carries an increased risk of dissection, rupture, or thromboembolic events [57].

Aortic Tumors: Secondary tumors are far more common than primary ones, usually resulting from direct invasion or hematogenous metastasis from malignancies such as lung or esophageal cancer [58]. Primary aortic tumors are exceedingly rare but highly aggressive, with a strong tendency toward arterial embolization and distant metastasis, leading to rapid clinical deterioration and poor survival outcomes [59, 60].

## 3. Diagnosis and Imaging of Aortic Diseases

### 3.1 Acute Aortic Syndrome (AAS)

The diagnosis of AAS remains clinically challenging due to its nonspecific and overlapping presentation with other life-threatening emergencies. A high index of suspicion is warranted when characteristic symptoms and signs are present (Table 2, Ref. [61]). Early recognition is critical, as delays in diagnosis significantly increase morbidity and mortality.

**Table 2.** **Signs and symptoms of AAS**.

Clinical signs and symptoms	
Asymmetric blood pressure (>20 mmHg) between limbs	Chest and back pain
Abdominal pain, gastrointestinal bleeding	Dyspnea or Shortness of breath
Hoarseness	Hemoptysis
Horner’s syndrome	Dysphagia
New murmur of aortic regurgitation	Oliguria or hematuria (gross)
Paraplegia	Lower extremity ischemia
Shock	Stroke symptoms
Superior vena cava syndrome	Syncope*

*Syncope is a particularly sinister symptom and may portend neurologic involvement or major cardiac dysfunction [61].

In patients exhibiting these clinical features, a targeted assessment of family history—including aortic aneurysm, aortic dissection, heritable aortopathy, or unexplained sudden death—is essential for risk stratification and identifying potential genetic predisposition. Clinical decision tools, such as aortic dissection risk scores, can assist in estimating the pre-test probability of AAS and guide further diagnostic evaluation [1]. Although an electrocardiogram (ECG) and chest X-ray may reveal abnormalities in some patients with aortic dissection, these findings are often non-specific and insufficient for definitive diagnosis [62]. While computed tomography (CT), transesophageal echocardiography (TEE), and MRI all exhibit high sensitivity and specificity for detecting AAS, contrast-enhanced CT angiography (CTA) of the entire aorta is the preferred initial imaging modality in suspected cases. This preference is based on its broad availability, rapid image acquisition, excellent spatial resolution, and unparalleled ability to provide comprehensive anatomical detail of the aortic wall, lumen, branch vessels, and surrounding structures [25, 63]. Importantly, when evaluation of the aortic root and ascending aorta is required, ECG-gated CTA should be performed to minimize motion artifacts caused by cardiac pulsation, thereby enhancing the accuracy of measurements and diagnostic confidence. In hemodynamically unstable patients or those with contraindications to iodinated contrast, either transthoracic or transesophageal echocardiography serves as a valuable alternative. TEE, in particular, offers high diagnostic accuracy for proximal aortic pathology and can detect complications such as pericardial effusion with tamponade physiology and severe aortic regurgitation. MRI, though highly accurate, is seldom employed in the acute setting due to prolonged scan times and limited accessibility; its principal utility lies in follow-up surveillance and in the evaluation of stable patients who cannot receive iodinated contrast. Currently, no circulating biomarker is diagnostic for AAS. However, D-dimer demonstrates a strong negative predictive value, making it a potentially useful tool for excluding AAS in low-to-intermediate risk patients when used in conjunction with clinical assessment [64].

### 3.2 Chronic Aortic Aneurysmal Diseases

A comprehensive history should focus on risk factors such as hypertension, smoking, and hypercholesterolemia, as well as a family history of aneurysms, congenital valvular heart disease, and autoimmune disorders. Most chronic aortic aneurysms remain asymptomatic for extended periods. Symptoms, such as chest/back pain, hoarseness, dyspnea, or dysphagia, typically arise from the mass effect on surrounding structures or an acute event [65, 66, 67]. Physical examination findings are frequently unremarkable; however, a palpable, pulsatile abdominal mass is a classic clinical sign of AAA. In patients with Marfan syndrome, characteristic phenotypic features may include tall stature, dolichostenomelia, arachnodactyly, ectopia lentis, and chest wall deformities such as pectus excavatum or carinatum [41]. For suspected aortic aneurysms, CTA extending from the carotid arteries to the femoral arteries is the first-line study and gold-standard diagnostic modality. It delivers high-resolution, three-dimensional visualization of the entire aorta and enables the detection of concomitant pathology, including coronary artery disease and branch vessel involvement [34]. MRI, which does not involve ionizing radiation, is particularly advantageous for evaluating congenital aortic anomalies and is the preferred technique for serial monitoring in younger patients and in clinical scenarios where radiation exposure must be minimized—such as during pregnancy or in genetically predisposed individuals requiring lifelong surveillance. Although transthoracic and TEE are limited in their ability to visualize the full extent of the aorta and therefore provide an incomplete anatomical assessment [1], these modalities remain essential components of pre-procedural cardiovascular evaluation, particularly for assessing left ventricular function, valvular pathology, and the risk for coronary artery disease prior to invasive interventions [68].

In contrast to AAS, the management of chronic aortic aneurysmal diseases places greater emphasis on early identification through targeted screening and long-term, dynamic surveillance rather than on urgent diagnosis. Currently, there are no universally endorsed population-based screening programs for thoracic aortic aneurysms. Nevertheless, evidence indicates that approximately 20% of patients with TAA have a first-degree relative affected by a similar condition, underscoring the value of genetic testing for pathogenic variants as a targeted screening strategy in high-risk families [69, 70]. Genetic testing plays a central role in the evaluation of syndromic heritable thoracic aortic disease (HTAD). The joint guidelines from the EACTS and the STS provide a structured diagnostic algorithm for genetic testing in HTAD and establish evidence-based diameter thresholds for surveillance across various aortopathy syndromes [1]. For abdominal aortic aneurysms, abdominal ultrasound is the recommended modality for both initial screening and ongoing surveillance due to its accessibility, cost-effectiveness, and safety profile [71].

### 3.3 Diagnosis of Other Aortic Conditions

Comprehensive imaging is essential for diagnosing less common aortic pathologies. Aortitis: 18F-FDG PET/CT (18F-fluorodeoxyglucose positron emission tomography/computed tomography)is the modality of choice for assessing active inflammation in vessel walls, particularly for Takayasu arteritis and giant cell arteritis, often revealing thickened walls and increased metabolic activity [51]. Infectious Aortitis: CT angiography (CTA) typically demonstrates periaortic fluid, soft tissue stranding, and occasionally gas bubbles or pseudoaneurysm formation [53]. Aortic Coarctation: MRI and CTA are critical for delineating the anatomy, measuring the pressure gradient indirectly through assessment of collateral vessels, and planning interventions [72]. Aortic Tumors: While rare, primary malignancies (e.g., angiosarcoma) require multi-modality imaging (MRI/CT) to characterize localized tissue invasion and metastatic spread [60].

## 4. Treatment of Aortic Diseases

### 4.1 Acute Aortic Syndrome

AAS encompasses a spectrum of life-threatening vascular emergencies that require prompt evaluation and immediate intervention to prevent catastrophic outcomes. Clinical management is primarily guided by the anatomical extent of disease and individual patient comorbidities. Regardless of the subtype, all patients with AAS must receive immediate initiation of optimal medical therapy (OMT), which serves as the foundation for any treatment strategy. OMT involves strict control of hemodynamic parameters—specifically targeting a systolic blood pressure of 100–120 mmHg and a heart rate of 60–80 beats per minute—along with effective pain management. Beta-blockers, frequently combined with intravenous vasodilators, constitute the cornerstone of antihypertensive treatment, while invasive arterial pressure monitoring is strongly recommended for critically ill patients in the intensive care unit [1, 73]. Opioid analgesics are indicated to mitigate pain-induced sympathetic activation, thereby reducing secondary hypertension and tachycardia [25]. Concomitant with medical stabilization, rapid assessment for definitive surgical or endovascular intervention is essential. Increasing evidence indicates that long-term adherence to oral antihypertensive therapy contributes to improved postoperative outcomes and a reduced risk of adverse aortic events in survivors of AAS [74, 75, 76].

#### 4.1.1 Acute Aortic Dissection

**Fig. 4.**
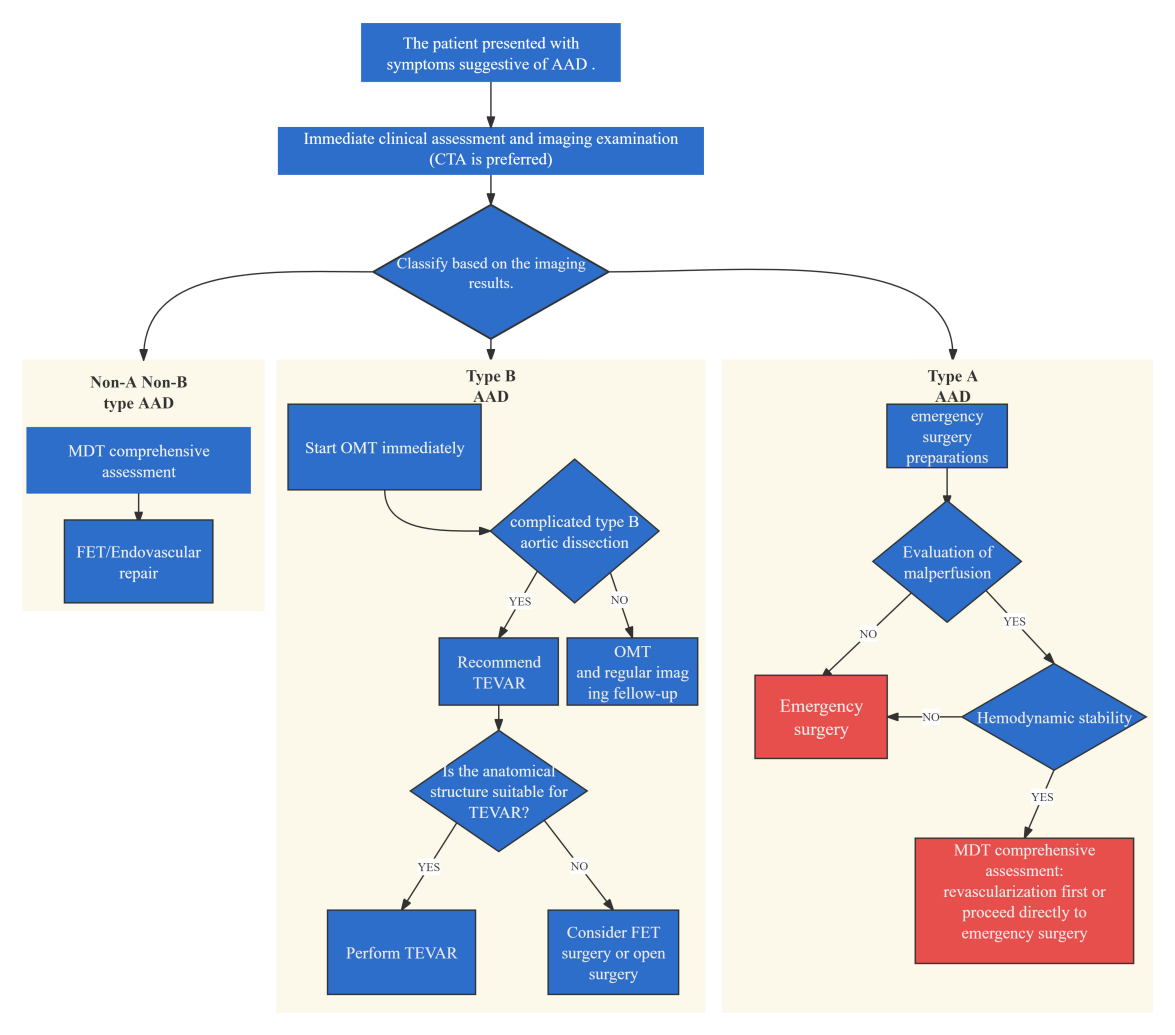
**Treatment strategy for acute aortic dissection**. CTA, computed tomography angiography; AAD, Acute aortic dissection; MDT, Multidisciplinary team; OMT, optimal medical treatment; TEVAR, Thoracic endovascular aortic repair; FET, frozen elephant trunk.

The principal objectives of open surgical or endovascular stent graft repair in acute aortic dissection are to prevent or manage aortic rupture and to halt the progression of the dissection flap by eliminating entry tears. Treatment strategies for aortic dissection are illustrated in Fig. 4.

Acute Type A Aortic Dissection: Surgical repair has been consistently associated with significantly lower mortality compared to medical management alone (with perioperative mortality rates ranging from 15% to 25% in large registries vs. >50% for medical therapy) and is therefore the standard of care unless contraindicated by severe comorbidities or prohibitive surgical risk [77]. Risk stratification is critical for individualized decision-making in ATAAD(Acute Type A Aortic Dissection). The GERAADA(German Registry of Acute Aortic Dissection Type A) score, originally introduced to predict 30-day mortality, has gained significant importance. The 2024 EACTS/STS Guidelines now assign a Class IIa recommendation for its use in patients undergoing surgery for ATAAD [1]. Recent validations have further solidified its utility; a systematic review and meta-analysis by Gemelli *et al*. [78] demonstrated that the GERAADA score outperforms the EuroSCORE II in predicting mortality specifically for ATAAD patients, highlighting the importance of disease-specific risk models over general cardiac surgery scores. In addition to GERAADA, the Penn classification provides a robust assessment based on the patient’s ischemic presentation. It categorizes patients into Class A (no ischemia), Class B (localized branch vessel malperfusion), and Class C (generalized ischemia or circulatory collapse). Multiple studies have verified the Penn classification as a strong independent predictor of operative mortality, emphasizing that the physiological impact of malperfusion is often the primary determinant of survival [79, 80]. To facilitate clinical application, Table 3 summarizes the key characteristics, advantages, and limitations of these two major risk assessment tools. Preoperative evaluation must prioritize the detection of malperfusion syndromes, as their presence correlates strongly with elevated perioperative mortality—reaching up to 43.4%—with risk increasing proportionally to the number of compromised organ systems [81]. Contemporary surgical management of Type A dissection has transitioned from a uniform approach to a precision medicine paradigm based on individualized anatomical and clinical assessment. Key principles include: first, tailoring the extent of repair—from isolated aortic root procedures to total arch replacement with the Frozen Elephant Trunk (FET) technique—based on dissection morphology, location of the primary entry tear, and patient-specific factors; second, adherence to a standardized operative protocol incorporating axillary artery cannulation, deep hypothermic circulatory arrest (DHCA) with antegrade cerebral perfusion, and an open distal anastomosis, all aimed at maximizing cerebral protection and ensuring durable distal sealing. While the FET technique has improved outcomes, demonstrating an operative mortality of approximately 8–15% and a spinal cord injury rate of 4–8% in contemporary series [1], it is not without complications. A significant issue is the occurrence of Distal Anastomotic New Entry (DANE) tears, which can promote false lumen patency. To address such limitations, recent trials have evaluated novel self-expanding, braided stents designed to be deployed in emergency conditions to treat malperfusion and stabilize the true lumen [82, 83]. Early long-term evidence suggests these evolving devices may offer promising solutions for high-risk anatomies. The overarching goal remains the definitive exclusion of the primary entry tear, with extension into the proximal descending aorta when necessary to promote true lumen re-expansion and false lumen thrombosis, ultimately improving long-term survival and aortic stability [84].

**Table 3.** **Comparison of key risk stratification scores for acute Type A aortic dissection**.

Scoring system	Primary focus/variables	Key advantages	Limitations/considerations
GERAADA score	Demographics & Anatomical complexity: Age, sex, malperfusion, arch involvement, Hemodynamic stability.	∙ Disease-specific: Designed exclusively for ATAAD.	∙ Requires detailed anatomical parameters derived from CT imaging.
		∙ Guideline Endorsed: Class IIa in 2024 EACTS/STS Guidelines.	∙ Calculation is more complex without the online tool.
		∙ Superior Accuracy: Outperforms EuroSCORE II in recent meta-analyses.	
Penn classification	Ischemic Presentation:	∙ Clinical Simplicity: Based on immediate physical signs and basic assessment.	∙ Does not account for detailed aortic anatomical complexity (e.g., tear location).
	∙ Class A: No ischemia.	∙ Strong Predictor: Directly correlates mortality with the severity of malperfusion.	∙ Qualitative categorization rather than a quantitative probability score.
	∙ Class B: Localized ischemia (e.g., limb, renal, visceral).		
	∙ Class C: Generalized ischemia/Circulatory collapse.		

EACTS, European Association for Cardio-Thoracic Surgery; STS, Society of Thoracic Surgeons; CT, computed tomography; ATAAD, Acute Type A Aortic Dissection; GERAADA, German Registry of Acute Aortic Dissection Type A.

Non-A-Non-B Aortic Dissection (Arch Involvement): Management strategies for dissections involving the aortic arch—with primary entry tears localized within this segment—are evolving from traditional open repair toward hybrid and endovascular techniques. While total arch replacement combined with FET may offer superior mid- to long-term outcomes, including improved survival and reduced reintervention rates compared to Thoracic Endovascular Aortic Repair (TEVAR) alone in select cohorts [85, 86], its increased procedural complexity and associated perioperative morbidity, particularly among elderly and high-risk patients, remain substantial limitations. These challenges have prompted innovation in hybrid approaches, such as supra-aortic debranching followed by TEVAR or direct arch vessel reconstruction with stent graft placement via median sternotomy, designed to achieve comparable therapeutic efficacy with lower initial procedural risk [87]. Currently, no universally accepted guidelines exist for the management of acute or subacute arch dissections. Future advancements are expected to focus on personalized treatment algorithms based on detailed anatomical classification and comprehensive risk profiling, thereby optimizing the balance between procedural safety and long-term effectiveness.

Acute Type B Aortic Dissection: The management of Type B dissection has shifted from a one-size-fits-all conservative model to a refined, risk-adapted strategy rooted in precision medicine. Central to clinical decision-making is the identification of high-risk features—both imaging and clinical—that predict complications such as malperfusion, rapid aortic expansion, or rupture. These include a large proximal entry tear (>10 mm), tear location along the inner curvature, unfavorable false lumen hemodynamics (e.g., patent false lumen, lack of thrombosis), and increased diameters of the aorta or false lumen [88, 89]. Risk stratification determines a clear therapeutic algorithm: in uncomplicated cases lacking these features, aggressive OMT combined with close radiological surveillance constitutes the standard of care. In contrast, patients presenting with complications (e.g., malperfusion, rupture, refractory pain) or high-risk characteristics should undergo early TEVAR. TEVAR is now established as the first-line intervention in such cases, effectively sealing the primary entry tear, restoring true lumen perfusion, and promoting favorable aortic remodeling, supported by robust clinical evidence that reports a 30-day mortality of 2–9% and a stroke rate of <5% for complicated cases [90, 91]. For patients with anatomical limitations precluding TEVAR—including inadequate proximal or distal landing zones, underlying connective tissue disorders, or retrograde extension into the arch—open surgical repair (e.g., total arch replacement with FET) remains a vital option, particularly in specialized centers with extensive experience in complex aortic surgery [92, 93]. Ongoing research should prioritize the integration of large-scale multicenter registry data to refine predictive models, optimize the timing for intervention, and guide individualized treatment selection [94].

#### 4.1.2 Intramural Hematoma

Type A Intramural Hematoma: The management of Type A intramural hematoma (IMH) follows a well-defined, risk-adapted strategy centered on the identification of high-risk clinical and imaging features. Established predictors of adverse outcomes include advanced age (>70 years), maximal aortic diameter exceeding 45 mm, hematoma thickness ≥10 mm, presence of pleural effusion, and ulcer-like projections [95, 96, 97]. The presence of one or more of these features is strongly associated with an elevated risk of disease progression, aortic rupture, or cardiovascular complications, and therefore warrants prompt surgical intervention [98]. In contrast, for hemodynamically stable patients without such high-risk characteristics, a conservative approach involving OMT under intensive surveillance may be cautiously adopted. However, patients must be thoroughly counselled regarding the potential for clinical deterioration necessitating delayed surgery. Serial imaging, preferably with CTA, should be performed at regular intervals to detect early signs of progression [99]. This risk-stratified framework enables individualized decision-making and highlights the critical importance of accurate, high-resolution imaging in guiding therapeutic strategies.

Type B Intramural Hematoma: Clinical management of Type B IMH is guided by a structured decision-making algorithm based on disease complexity. In complicated cases—defined by evidence of aortic expansion, impending rupture, malperfusion, or persistent pain—emergency intervention is indicated, with TEVAR established as the first-line treatment due to its minimally invasive nature and favorable short-term outcomes. For uncomplicated Type B IMH, initial management consists of strict blood pressure control through optimal medical therapy and close radiological monitoring, recognizing that even clinically stable presentations can evolve into life-threatening complications over time [96, 100]. Two key challenges remain in current practice: first, the precise identification of patients at high risk of failure with medical therapy alone who may benefit from early intervention; and second, determining the optimal timing of intervention, as emerging data suggest that deferred TEVAR—performed after initial stabilization—may yield improved long-term results compared to immediate repair [101]. In anatomically complex cases unsuitable for endovascular repair or in the presence of specific underlying pathologies, open surgical repair remains a vital salvage option, particularly in specialized centers with expertise in aortic surgery.

#### 4.1.3 Penetrating Atherosclerotic Ulcer

The management of penetrating atherosclerotic ulcers (PAUs) is guided by well-established principles of risk stratification and anatomical localization. Central to clinical decision-making is the identification of high-risk features, as detailed in Table 4 (Ref. [97, 102, 103, 104, 105, 106, 107, 108]), which constitute the primary indications for intervention. For high-risk PAUs involving the ascending aorta, open surgical repair remains the treatment of choice due to the proximity to critical structures such as the aortic valve and coronary ostia, as well as the elevated risk of rupture. In contrast, for high-risk PAUs located in the descending thoracic aorta, TEVAR is the standard of care, offering effective lesion exclusion with favorable procedural outcomes and reduced perioperative morbidity. Progress in endovascular technology has expanded therapeutic options for complex anatomical presentations: in cases of arch involvement where conventional TEVAR is not feasible because of inadequate landing zones, hybrid procedures such as the FET technique or the use of customized fenestrated or scalloped stent grafts provide viable and effective alternative strategies [109]. Given the distinct clinical profile of patients with PAUs—typically elderly individuals with extensive comorbidities—a balanced management approach is essential. Conservative management with close surveillance is appropriate for asymptomatic, low-risk lesions, while timely intervention is warranted in high-risk cases. This dual strategy underscores a patient-centered philosophy that emphasizes individualized risk assessment and tailored therapeutic planning.

**Table 4.** **High-risk features in aortic intramural hematoma and penetrating atherosclerotic ulcer**.

Risk category	High risk feature	Applicable condition
Morphologic criteria	Pleural effusion (based on Hounsfield units)	IMH [97, 102], PAU [103, 104]
	Aortic ulcer/Ulcer-like projection	IMH [105, 106]
	Presence of intramural hematoma	PAU [103]
	Initial aortic diameter >45 mm	IMH [107]
	Wall thickness of involved segment ≥10 mm	IMH [108]
	Large initial PAU depth (>10 mm) and diameter (>20 mm) or high growth rate	PAU [103]
	Mean aortic diameter growth rate ≥5 mm/year	IMH [107]
Clinical criteria	Age >70 years	IMH [105]
	Persistent pain despite medical treatment	PAU [103]

Notes: IMH, Intramural Haematoma; PAU, Penetrating Atherosclerotic Ulcer.

#### 4.1.4 Traumatic Aortic Injury

Penetrating trauma typically results in direct aortic wall disruption and necessitates emergency surgical intervention for hemorrhage control. In contrast, blunt traumatic aortic injury—though less immediately catastrophic—is more prevalent and requires prompt management guided by Advanced Trauma Life Support (ATLS) protocols, with definitive diagnosis achieved through CTA. Following the confirmation of an injury, treatment decisions are strictly determined by the established injury grading system. Hemodynamically stable Grade I injuries and select Grade II injuries without high-risk imaging features are managed non-operatively, involving strict hemodynamic control (target blood pressure and heart rate parameters) and serial imaging surveillance to monitor for progression. For all Grade III and IV injuries, as well as Grade II injuries exhibiting high-risk characteristics—such as mediastinal hematoma exceeding 10 mm or a lesion-to-normal aortic diameter ratio greater than 1.4, TEVAR is recommended as the first-line intervention when anatomical criteria are met [25, 110, 111]. To ensure optimal perioperative outcomes, transfer to high-volume centers with specialized expertise in vascular trauma is strongly advised [112].

### 4.2 Chronic Aortic Aneurysmal Diseases

#### 4.2.1 Thoracic Aortic Aneurysms

Root and Ascending Aorta: The primary objective of surgical intervention for aortic root aneurysms is to eliminate the risk of rupture or dissection while preserving and appropriately managing aortic valve function. In patients with morphologically suitable valve leaflets, valve-sparing root replacement (e.g., the David procedure) is the preferred approach, as it avoids lifelong anticoagulation and demonstrates excellent long-term durability and valve preservation [113, 114, 115]. Conversely, for patients with irreparable aortic valves or specific genetic connective tissue disorders such as the Loeys-Dietz syndrome, the Bentall procedure remains the gold standard [116, 117]. In cases where aneurysmal dilation is limited to the tubular portion of the ascending aorta with a normal aortic root, isolated supra-coronary ascending aortic replacement is the standard surgical intervention; the preserved native root typically demonstrates slow expansion and a low incidence of late reintervention [118]. The selection of surgical technique depends on multiple factors, including aneurysm morphology, aortic valve integrity, patient age, comorbidities, and institutional surgical expertise.

For chronic ascending aortic aneurysms, the operative strategy is determined by the anatomical extent of disease involvement. Isolated replacement of the tubular ascending aorta is indicated for segmentally confined pathology. When the lesion extends into the proximal aortic arch, hemiarch replacement performed under DHCA is required to achieve adequate resection margins. However, evidence indicates that extending resection to include the arch in the absence of a true arch aneurysm—solely for the purpose of a “more radical” approach—may increase procedural risk without clear benefit, and therefore should be carefully evaluated on a case-by-case basis [119]. For more extensive disease involving the mid-arch, partial arch replacement with supra-aortic vessel reimplantation may be employed. A key principle in contemporary surgical planning is future-proofing the aorta: implantation of a sufficiently long (≥7 cm) straight graft during the initial repair provides an optimal proximal landing zone for potential future endovascular interventions targeting descending aortic pathology.

Aortic Arch: Pathologies involving the aortic arch frequently extend into adjacent segments, necessitating a comprehensive, multidisciplinary approach to decision-making. Open total arch replacement combined with the FET technique represents a definitive solution for complex aneurysms with distal aortic involvement; however, due to its high procedural complexity and associated morbidity, careful patient selection is imperative [120, 121]. As a risk-mitigation strategy, hybrid procedures—such as supra-aortic debranching followed by TEVAR—have emerged as a valuable alternative for high-risk surgical candidates, although they carry distinct complications, including an elevated risk of stroke [122]. Emerging branched endovascular repair technologies are expanding treatment options for patients previously deemed inoperable. While long-term data on durability and outcomes remain under investigation, these innovations represent an important advancement in the evolution of arch-directed therapies [123]. Consequently, modern management of aortic arch disease encompasses a therapeutic spectrum, requiring individualized selection among open surgical, hybrid, and fully endovascular strategies based on anatomical configuration, patient-specific risk factors, and center-specific capabilities.

Descending Aorta: TEVAR is the first-line treatment for descending thoracic aortic aneurysms and related pathologies, owing to its minimally invasive nature and favorable short- and medium-term safety profile, with perioperative mortality rates typically <2% for elective TEVAR compared to 5–10% for open repair [124, 125, 126]. The procedural goal is exclusion of the diseased segment through precise stent-graft deployment, leading to thrombosis of the aneurysm sac. In Type B aortic dissection (TBAD), successful TEVAR involves sealing the proximal entry tear, thereby promoting false lumen thrombosis, true lumen expansion, and favorable aortic remodeling—all contributing to improved clinical outcomes. Technical success in TEVAR hinges on two critical factors: first, the proximal landing zone must provide adequate length (>25 mm) of healthy aortic tissue and meet quality criteria—including absence of connective tissue disease, diameter ≤38 mm, and minimal mural thrombus or calcification—to minimize risks such as type Ia endoleak and retrograde Type A dissection [127]; second, stent-graft oversizing must be tailored to the underlying pathology: typically 15–20% for chronic aneurysms, but restricted to <10% using low-radial-force devices in acute or subacute dissections due to the vulnerability of the aortic wall [128, 129]. In cases of inadequate landing zones, open surgical intervention, particularly the FET technique, is the primary option to establish a durable seal. Conventional open repair remains essential for managing TEVAR failure, anatomical contraindications, or concomitant conditions such as mycotic aneurysm or active infection [130].

#### 4.2.2 Thoracoabdominal Aortic Aneurysms

The management of TAAAs is characterized by the parallel advancement of open surgical and endovascular techniques, each offering distinct advantages and challenges. Classic open repair remains a highly complex intervention, with procedural success critically dependent on comprehensive organ protection strategies. These include the use of partial cardiopulmonary bypass or left heart bypass to maintain systemic perfusion, implementation of a sequential cross-clamping technique to preserve distal blood flow, and meticulous reimplantation or preservation of intercostal and visceral arteries to reduce the risk of spinal cord ischemia—a major cause of postoperative morbidity [131, 132]. A key contemporary surgical principle is the “distal-first” approach, in which a prior FET or TEVAR establishes a stable proximal landing zone. This strategy simplifies subsequent open repair, minimizes manipulation of the left lung, and may contribute to improved perioperative outcomes [133]. Concurrently, branched and fenestrated endovascular aortic repair (B/FEVAR) has emerged as a first-line therapeutic option for the majority of TAAAs, particularly in anatomically suitable patients, owing to its minimally invasive nature and favorable short-term outcomes [134, 135]. B/FEVAR utilizes a custom-designed main stent-graft featuring branches or fenestrations that align with target visceral and renal arteries, allowing deployment of bridging stent-grafts to maintain perfusion while achieving complete aneurysm exclusion. Branched configurations are typically favored for longer distances between the main body and target vessels, whereas fenestrated devices enable more distal sealing zones, potentially preserving additional intercostal arteries and lowering the risk of paraplegia [136, 137]. Although B/FEVAR significantly reduces perioperative mortality, major complications, and hospital length of stay, it is associated with a higher long-term reintervention rate, primarily due to complications involving the bridging stent-grafts—most commonly renal artery stent thrombosis or type III endoleaks [138, 139].

#### 4.2.3 Abdominal Aortic Aneurysms

For infrarenal abdominal aortic aneurysms with adequate proximal neck anatomy (defined as neck length >10 mm), EVAR is the preferred initial treatment modality, largely due to its substantially lower perioperative mortality rate (approximately 1.6%) compared to open repair [140, 141]. However, EVAR is associated with a higher incidence of long-term secondary interventions, making open surgical repair a viable and often preferable alternative for younger, low-risk patients with extended life expectancy. Optimal treatment selection requires rigorous preoperative assessment of landing zone characteristics. EVAR typically necessitates 10–25% stent-graft oversizing to ensure secure fixation and seal; adjunctive techniques such as coil embolization of the inferior mesenteric artery or the use of iliac branch devices (IBDs) can be employed to mitigate the risk of type II endoleak and preserve pelvic arterial perfusion [142, 143]. In patients with challenging neck anatomy—including short necks (5–10 mm) or juxtarenal aneurysms (<5 mm)—the two principal management strategies are open surgical repair via a retroperitoneal or transperitoneal approach with supraceliac or suprarenal aortic control, and B/FEVAR. B/FEVAR offers lower rates of early complications and shorter recovery times but entails a greater likelihood of mid- to long-term reinterventions. The optimal choice depends on multiple factors, including aneurysm morphology, patient comorbidities, life expectancy, and institutional expertise in complex endovascular procedures [144, 145]. In the setting of ruptured abdominal aortic aneurysms (rAAAs), EVAR—when anatomically feasible—confers significant benefits, including reduced intensive care unit (ICU) and overall hospital length of stay, with particular advantages observed in elderly patients and females. Nevertheless, long-term survival outcomes appear comparable between EVAR and open surgical repair, with overall mortality remaining high at approximately 30–50% regardless of the chosen modality [146]. Furthermore, mycotic (infected) aortic aneurysms require urgent surgical intervention irrespective of size, and are always accompanied by prolonged antibiotic therapy. While open repair remains the standard of care, the role of EVAR is expanding, with growing evidence indicating reduced short-term mortality, albeit at the cost of higher infection-related reintervention rates [147]. For saccular aneurysms, which are considered to carry a higher rupture risk than fusiform lesions, a lower threshold for intervention is warranted. EVAR is increasingly utilized in these cases and has demonstrated technical feasibility and favorable mid-term durability [148]. Isolated abdominal aortic dissections are rare clinical entities. Current limited evidence suggests that EVAR may be associated with lower mortality and fewer major complications compared to conservative management, although further research is needed to establish definitive guidelines [149].

#### 4.2.4 Iliac Artery Aneurysms

In the management of iliac artery aneurysms, preservation of antegrade flow in at least one internal iliac artery (IIA) has emerged as a fundamental principle. This strategy significantly reduces the risk of postoperative complications, including buttock claudication, colonic ischemia, pelvic necrosis, and sexual dysfunction. The IIA serves as a critical collateral pathway for spinal cord perfusion, and maintaining its patency is particularly important in patients undergoing extensive aortic interventions to minimize the risk of paraplegia [150, 151, 152]. For iliac aneurysms with a diameter ≥35 mm, endovascular repair is associated with lower perioperative mortality and complication rates compared to open surgical repair in both elective and ruptured scenarios [153]. Among available endovascular techniques, the iliac branch stent (IBS) device—now commercially available and regulatory-approved—represents a preferred option. It enables effective aneurysm exclusion while preserving IIA perfusion, demonstrating high technical success and patency rates, and significantly reducing the incidence of buttock claudication when compared to the conventional approach of IIA embolization followed by stent-graft extension into the external iliac artery (EIA) [154]. When unilateral IIA embolization is necessary, occlusion of the proximal main trunk is favored over selective distal branch embolization, and the contralateral IIA must remain patent to preserve collateral circulation and mitigate ischemic complications [150, 151]. In approximately 40% of patients with concomitant a AAA and a common iliac artery aneurysm, the IBS technique can establish an optimal distal landing zone for EVAR; conversely, EVAR may create a suitable proximal landing zone for isolated IBS placement, illustrating a synergistic relationship between these two modalities [155]. Off-label endovascular strategies such as the “sandwich” technique may also be employed for IIA preservation but carry a risk of “gutter” endoleak; their long-term efficacy and safety require further validation through prospective studies [156, 157].

### 4.3 Other Aortic Conditions

This section addresses a spectrum of relatively rare yet clinically significant aortic pathologies that demand individualized, pathology-specific management strategies tailored to patient characteristics.

Aortic Graft/Stent Infection: This represents a severe and potentially life-threatening complication. Its management involves radical debridement, complete excision of infected prosthetic material, and durable revascularization. In-situ reconstruction at the anatomical site is recommended, utilizing bioprosthetic materials such as cryopreserved allografts, autologous vein conduits, surgeon-modified bovine pericardial grafts, or antibiotic-bonded synthetic grafts, frequently augmented with vascularized tissue flaps (e.g., omental flap) to enhance resistance to reinfection. For hemodynamically unstable patients, staged endovascular repair (TEVAR/EVAR) may serve as a temporizing bridge to definitive surgery, although it is associated with a high risk of persistent or recurrent infection [158, 159]. Long-term antibiotic therapy constitutes the cornerstone of treatment, typically administered for a minimum of six weeks postoperatively; in inoperable cases, lifelong suppressive therapy may be required [160].

Kommerell’s Diverticulum: Clinical decisions are guided by symptom status and aneurysm size. Surgical intervention is clearly indicated for symptomatic lesions or those exceeding 50–55 mm in diameter. Open surgical repair—with concomitant subclavian artery transposition or revascularization—is the standard of care for young, low-risk patients, offering definitive decompression and durable long-term results. Hybrid or total endovascular approaches—such as TEVAR combined with surgical bypass or the use of branched stent-grafts—provide minimally invasive alternatives for elderly or high-risk individuals, though they may entail an increased incidence of reintervention [57, 161, 162, 163, 164].

Aortic Coarctation: The primary therapeutic goal is relief of luminal obstruction. In adults and adolescents, balloon-expandable covered stent implantation has become the first-line intervention, effectively reducing pressure gradients and minimizing the risk of post-procedural aneurysm formation. Open surgical correction—including end-to-end anastomosis, patch aortoplasty, or bypass grafting—remains essential in pediatric patients, those with complex anatomy, or cases of restenosis following prior intervention [55, 165].

Inflammatory AD: The management of conditions such as Takayasu arteritis, giant cell arteritis, and infectious aortitis adheres to a triad of principles: pharmacologic control of inflammation or infection, selective intervention for structural complications, and lifelong imaging surveillance. During the active inflammatory phase, medical therapy takes precedence—corticosteroids and immunosuppressive agents for non-infectious vasculopathies, or targeted antimicrobial therapy for infectious aortitis. Surgical or endovascular intervention is reserved for complications, including aneurysm progression, rupture, critical stenosis, or refractory symptoms, and should ideally be performed during periods of disease quiescence [25, 166, 167]. In infectious aortitis (mycotic aneurysm), open surgical repair combined with prolonged antibiotic therapy remains the gold standard; endovascular repair is limited to palliative or bridging indications in high-risk, inoperable patients [147].

## 5. Conclusion

This review provides a systematic overview of the diagnosis and management of AD. Despite substantial advancements in imaging modalities, medical therapies, open surgical techniques, and endovascular interventions, the overall evidence in aortic medicine remains limited, with most clinical recommendations derived from small cohort studies and expert consensus. This reflects the inherent challenges of conducting robust research in a field defined by rare, acute, and life-threatening conditions requiring multidisciplinary coordination. To advance the field toward more precise, standardized, and evidence-based practice, future efforts must prioritize the resolution of key knowledge gaps.

Strengthening the Evidence Base: There is an urgent need for well-designed, multicenter, prospective randomized controlled trials (RCTs) and large-scale international registries to generate higher-level evidence, particularly regarding optimal surgical strategies and the timing of interventions.

Advancing Diagnostic and Monitoring Technologies: The identification and validation of specific biomarkers for early detection and risk stratification of acute aortic syndromes are essential. Additionally, the development of automated, Artificial Intelligence (AI)-assisted tools for aortic measurement can reduce interobserver variability and standardize longitudinal assessment.

Optimizing and Standardizing Treatment Strategies: Defining the optimal extent of surgical repair in Type A aortic dissection is crucial to balance comprehensive disease management against procedural morbidity. The role and long-term outcomes of endovascular therapies in patients with heritable thoracic aortic diseases warrant rigorous evaluation. Furthermore, standardized reporting of intraoperative parameters—such as duration and temperature during circulatory arrest at various anatomical levels—is imperative to enable meaningful comparisons across surgical series.

Implementing Standardized and Individualized Lifelong Management: The development and validation of disease- and modality-specific lifelong imaging surveillance protocols (e.g., post-TAAD, post-TBAD, post-EVAR, post-FET) are essential for timely detection of complications. Concurrently, research into the biological mechanisms underlying aortic wall regeneration and repair may pave the way for novel regenerative and biologic therapies.

In summary, through sustained collaboration within the global aortic community and a focused commitment to addressing these core challenges, current clinical dilemmas can be progressively resolved. Such efforts will deepen our understanding of aortic pathobiology, facilitate earlier diagnosis and intervention, and refine long-term management strategies, ultimately improving patient outcomes and long-term prognosis.

## Abbreviations

AAS, Acute Aortic Syndrome; AD, Aortic Disease; EACTS, European Association for Cardio-Thoracic Surgery; STS, Society of Thoracic Surgeons; SVS, Society for Vascular Surgery; ESVS, European Society for Vascular Surgery; CTA, Computed Tomography Angiography; MRI, Magnetic Resonance Imaging; BTAI, Blunt Traumatic Aortic Injury; TAA, Thoracic Aortic Aneurysm; TAAA, Thoracoabdominal Aortic Aneurysm; AAA, Abdominal Aortic Aneurysm; LDS, Loeys-Dietz Syndrome; vEDS, Vascular Ehlers-Danlos Syndrome; BAV, Bicuspid Aortic Valve; ECG, Electrocardiogram; TEE, Transesophageal Echocardiography; EVAR, Endovascular Aneurysm Repair; HTAD, Heritable Thoracic Aortic Disease; OMT, Optimal Medical Therapy; TEVAR, Thoracic Endovascular Aortic Repair; DHCA, Deep Hypothermic Circulatory Arrest; FET, Frozen Elephant Trunk; IMH, Intramural Hematoma; PAU, Penetrating Atherosclerotic Ulcer; ATLS, Advanced Trauma Life Support; B/FEVAR, Branched/Fenestrated Endovascular Aortic Repair; IIA, Internal Iliac Artery; EIA, External Iliac Artery; IBS, Iliac Branch Stent; ICU, Intensive Care Unit; RCT, Randomized Controlled Trial; AI, Artificial Intelligence; TTE, Transthoracic Echocardiography.
